# Preferred Sources of Health-Risk Information About Cigarettes, e-Cigarettes, and Waterpipes Among Adolescents in Germany: A Cross-Sectional Analysis of a Large School-Based Survey

**DOI:** 10.1177/1179173X261473915

**Published:** 2026-08-07

**Authors:** Stephanie Klosterhalfen, Cynthia Fedler, Reiner Hanewinkel, Clemens Neumann, Julia Hansen

**Affiliations:** 1 Institute of General Practice (ifam), Centre for Health and Society (chs), Addiction Research and Clinical Epidemiology Unit, Medical Faculty and University Hospital Düsseldorf, 9170 Heinrich Heine University, Düsseldorf, Germany; 2 221536 Institute for Therapy and Health Research, IFT-Nord, Kiel, Germany

**Keywords:** adolescent nicotine use, tobacco prevention, e-cigarettes, waterpipe, health communication, information avoidance, Germany, school-based survey

## Abstract

**Background:**

Adolescent nicotine use remains a major public health concern, particularly in the context of increasing e-cigarette uptake and gaps in tobacco-control communication. Effective prevention requires evidence on the information channels through which adolescents prefer to receive risk information about tobacco and nicotine products.

**Objectives:**

This study examined adolescents’ preferred sources for receiving information about the health risks of cigarettes, e-cigarettes, and waterpipes in Germany, and assessed differences by age, gender, school type, and nicotine-use status.

**Design:**

Cross-sectional analysis of a large nationwide school-based survey across 14 of 16 German federal states.

**Methods:**

Data were drawn from the 2024/2025 wave of the Präventionsradar study and included 21,841 adolescents aged 9–17 years from 116 schools and 1,712 classes across 14 German federal states. Weighted descriptive analyses and multilevel logistic regression models were used to examine preferred information sources and information refusal. Models were adjusted for age, gender, school type, and current nicotine-product use, with random intercepts at the class level.

**Results:**

Parents (30.2%), social media (21.5%), and physicians (18.6%) were the most frequently selected information sources. Younger adolescents more often selected parents, whereas older adolescents more often selected social media and peers. Adolescents currently using at least one tobacco or nicotine product were more likely to select friends and less likely to select parents, teachers, or physicians. They were also more likely to report not wanting health information. Information refusal was additionally associated with male gender and lower academic track.

**Conclusion:**

Adolescents’ preferred sources of tobacco- and nicotine-related health information differ substantially by sociodemographic characteristics and nicotine-use status. These findings may inform the development of differentiated and multimodal communication strategies.

## Introduction

Globally, adolescent nicotine use remains a persistent public health concern. While overall tobacco use has declined in many settings, the World Health Organization reports persistently high numbers of young users and growing uptake of e-cigarettes among adolescents worldwide.^1^ The health risks of nicotine use are well established, and adolescents are particularly vulnerable because of ongoing neurobiological and psychosocial development.^2,3^ The rapid uptake of e-cigarettes and other non-combustible nicotine products among young people further raises concern, particularly in light of emerging evidence that nicotine itself may exert direct cardiovascular toxicity, independent of combustion.^4^ To prevent nicotine dependence and related harms, prevention — alongside regulation, structural measures such as smoke-free policies, and cessation support — remains essential. In this context, understanding adolescents’ information environments has become increasingly relevant, particularly the sources and channels through which they prefer to receive such information.

In Germany, tobacco-control policies remain comparatively weak by international standards, with Germany ranked 34th out of 36 countries on the Tobacco Control Scale in 2021. Particularly notable is the absence of substantial public funding for national mass-media campaigns, as reflected by a score of 0 in the “Spending on public health information campaigns” category. This suggests structural gaps in population-wide communication efforts.^5^ At the same time, the nicotine-product landscape has changed considerably. Recent German data indicate a marked shift toward e-cigarettes and the emergence of nicotine pouches, while waterpipe use is declining and heated tobacco products remain rare among adolescents.^6^ In 2023, among 12–17-year-olds in Germany, current use was 7.4% for cigarettes, 6.7% for disposable e-cigarettes, and 3.9% each for reusable e-cigarette use and waterpipe use.^7^ Given this evolving product landscape, strategic selection of messengers and communication channels is critical for adapting prevention efforts to adolescents’ needs and media environment.

Effective tobacco and nicotine prevention also depends on whether adolescents are willing to receive credible information and through which sources they prefer to receive it. Previous research points to several sources: clinicians, parents, schools, on-pack warnings, peers, and social media. Qualitative studies suggest that physicians and package warnings are often perceived as trustworthy, while peers and online media play a prominent role in everyday exposure to tobacco- and nicotine-related information.^8,9^ Experimental studies further show that messages from expert sources are rated as more credible and may increase adolescents’ perceptions of the harmfulness of tobacco and nicotine products compared with messages from peers or influencers.^10,11^ Thus, adolescents’ preferred information sources are not only a descriptive feature of their information environment, but may be relevant for the design and delivery of prevention messages.

Consistent with this, one study analyzing reported information sources observed that monitored sources, such as school, parents, and mass media, were negatively associated with adolescent tobacco use, whereas unmonitored sources, such as peers, siblings, and the internet, were positively associated with use.^12^ However, existing evidence is predominantly international, and findings may not be directly transferable to the German context because of differences in school systems, media use, and tobacco-control regulation. Evidence on adolescents’ preferred sources of tobacco- and nicotine-related health information in Germany remains limited, particularly with regard to differences by nicotine-use status, school type, age, and gender.

Identifying adolescents’ preferred sources of tobacco- and nicotine-related risk information is relevant for tobacco control because prevention messages can only be effective if they reach the intended audiences through credible and acceptable channels. This is particularly important for adolescents who already use nicotine products, as they may be less receptive to conventional school- or parent-based prevention. Against this background, this study aimed to examine whether and how adolescents in Germany wish to receive information about the health risks of cigarettes, e-cigarettes, and waterpipes. Specifically, it examined preferred information sources and channels, differences by gender, age, school type, and nicotine-use status, and characteristics associated with refusal of health information. The study thereby contributes to the literature on adolescent tobacco and nicotine prevention by providing target-group-specific evidence for communication strategies in Germany.

## Materials and Methods

### Study Design and Data Source

The present study is based on data from the ninth wave of the Präventionsradar (https://www.praeventionsradar.de), a large-scale, school-based survey conducted using an online questionnaire among students in grades 5 through 10. Data collection took place between November 2024 and February 2025 across 14 German federal states. The survey aims to monitor health-related behaviors and perceptions among children and adolescents.

All schools in the participating federal states were invited to participate by email. In schools that agreed to participate, entire classes were invited to participate, with inclusion criteria requiring students to be enrolled in grades 5–10.

Informed consent was obtained in advance using written consent forms distributed to students and their parents or legal guardians by the school staff. The age threshold for independent student consent versus parental consent was determined in accordance with the specific legal regulations of the respective federal state. On the day of the survey, the voluntary nature of the study was re-emphasized to the students. Regardless of any prior written consent provided by parents or the students themselves, all participants retained the right to decline participation at any time on the day of data collection without giving reasons.

Data were collected using a structured online questionnaire covering demographic characteristics, tobacco and nicotine use, and preferences for information channels regarding tobacco and nicotine products.

The study followed ethical standards for research involving minors. Approval was obtained from the relevant educational authorities and the Ethics Committee of the German Society for Psychology on June 15^th^, 2016 (AZ RH 042015_1).

The reporting of this cross-sectional study follows the Strengthening the Reporting of Observational Studies in Epidemiology (STROBE) guidelines.

### Primary Endpoint

The primary endpoint was adolescents’ preferred sources for receiving information about the health risks of tobacco and nicotine products. Participants were asked: “How would you like to learn more about the health risks of waterpipes, cigarettes, or e-cigarettes? You may select multiple answers.” Response options included apps or websites, physicians, teachers, parents, friends, posters in public spaces, health insurance providers, sports clubs, and social media platforms such as TikTok, Instagram, and YouTube. Additional options were “other sources” and “I do not want to receive any information.” All options were presented in randomized order, except for the final two response options, which appeared in fixed positions. Each response option was coded as a binary variable (1 = selected, 0 = not selected). Participants could select multiple sources, allowing for overlapping preferences across categories. The item assessing preferred information sources had previously been used in a survey on waterpipe use [https://osf.io/347kc/overview]. Before its use in that survey, the questionnaire was pretested with adolescents and young adults aged 15-34 years to assess comprehensibility. For use in the Präventionsradar survey, selected questionnaire items were deliberately phrased in simpler and more age-appropriate language. However, the item on preferred information sources was not explicitly pretested among the full target population of students aged 9-17 years. Because the item was designed as a single multiple-response question and the response options represented distinct, non-mutually exclusive information preferences, measures of internal consistency were not applicable. Formal criterion validity and test–retest reliability were not assessed.

### Secondary Endpoints

Secondary analyses examined differences in preferred information sources by gender, age, school type, and nicotine-use status. In addition, participants who selected “I do not want to receive any information” were compared with those who selected at least one information source with respect to demographic characteristics, school type and nicotine-use status.

### Covariates

Covariates included age, gender, school type, and current nicotine-use status. Age was categorized as dichotomous variable (9–14 years and 15–17 years) to capture the distinct development shifts between early and middle adolescence, a classification well-established by the Lancet Commission on Adolescent Health and Wellbeing.^13^ School type was coded as Gymnasium, representing the highest academic track in Germany, versus other general educational school types. Current nicotine-product use was defined as the use of at least one of the assessed products – cigarettes, e-cigarettes, or waterpipe – at least once a month.

Current cigarette and e-cigarette use were assessed with the following questions, “How often do you currently smoke cigarettes?” and “How often do you currently use e-cigarettes?” Current waterpipe use was assessed separately with the question, “How often do you currently use a waterpipe?” Response options for all three questions were “Not at all,” “Less than once a month,” “At least once a month, but not every week,” “At least once a week, but not every day,” and “(almost) every day.” For the analysis, the responses were dichotomized: participants reporting use at least once a month were classified as current users, while those reporting “Not at all” or “Less than once a month” were classified as non-users. These covariates were included to adjust for potential confounding and to examine subgroup differences in preferred information sources.

Smoking in the home environment was assessed with the question, ‘Does anyone smoke at your home? This includes cigarettes, e-cigarettes, waterpipes, or any other use of smoking products.’ Response options were ‘no,’ ‘yes, including inside the apartment/house,’ and ‘yes, but only outside the apartment/house.’ Household smoking was included as an additional covariate in sensitivity analyses.

### Sample

All eligible schools were invited to participate via email, resulting in the initial registration of 2,009 classes. A total of 26,586 adolescents aged 9 to 17 years participated in the ninth wave of the study, drawn from 1,712 classes across 116 schools, representing a class-level participation rate of 85.2%.

**Figure 1.**
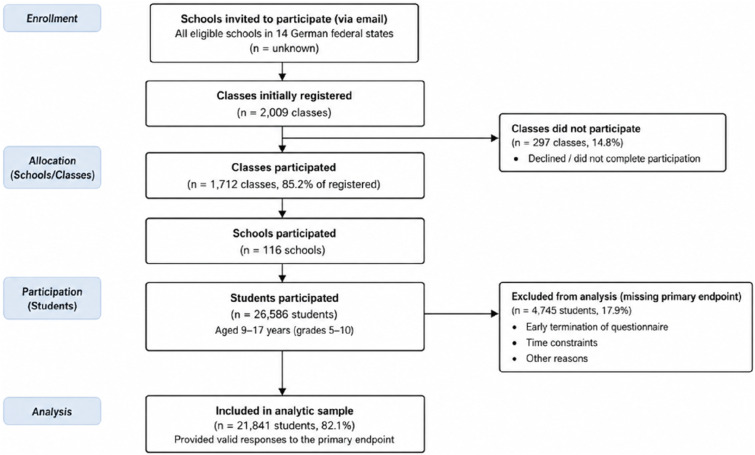
Flow chart – Präventionsradar (Wave 9)

Of the total sample, 21,841 participants provided valid responses to the primary endpoint and formed the analytic sample. The remaining 4,745 participants had missing data for the primary endpoint, mostly because of early termination of the questionnaire, time constraints, or other reasons (see Figure 1).

The mean age of the analytic sample was 13.3 years (SD = 1.76). Overall, 28.1% were aged 15-17 years, and 71.9% were aged 9-14 years; 47.6% were male. In total, 57.3% attended a Gymnasium, while the remaining participants attended other types of general-education schools found across the federal states, such as comprehensive schools, district schools, regional schools, and secondary schools. Current use of at least one assessed tobacco or nicotine product was reported by 9.1% of participants.

### Statistical Analyses

Preferences for information sources were analyzed using weighted descriptive statistics, with weights applied for age, gender, and school type according to population distributions provided by the Federal Statistical Office of Germany.

Associations between preferred information sources and demographic characteristics and behavioral factors were examined using multilevel logistic regression models. Separate models were estimated for each information source as a binary outcome. Models included age group, gender, school type, and nicotine-use status as fixed effects and a random intercept at the class level to account for clustering. Information refusal was analyzed as a binary outcome using multilevel logistic regression with the same covariates and random-intercept structure. Interaction terms between key sociodemographic variables and nicotine-use status were tested by including the corresponding interaction term in the multilevel logistic regression models. Adjusted odds ratios (aORs) with corresponding 95% confidence intervals (CIs) are reported. Statistical analyses were performed using Stata version 19.0. Statistical significance was set at p < 0.05.

Because the primary endpoint allowed multiple responses, selected information sources were not mutually exclusive. Results should therefore be interpreted as preferences for individual sources rather than as exclusive channel choices.

To assess the potential confounding effect of smoking in the home environment, sensitivity analyses were conducted by additionally adjusting the multilevel logistic regression models for household smoking.

## Results

### Preferred Sources of Health Risk Information

The most frequently reported sources of information about the health risks of waterpipes, cigarettes, and e-cigarettes were parents (30.2%), social media (21.5%), and physicians (18.6%).

Age-specific differences were observed. Younger adolescents aged 9-14 years were more likely to select parents as a preferred information source (32.8%) than older adolescents aged 15–17 years (23.6%). In contrast, older adolescents selected social media more frequently (26.9%) than younger adolescents (19.4%). Other sources showed smaller differences between age groups.

Girls were more likely than boys to select parents (31.8% vs. 28.7%), teachers (20.2% vs. 16.3%), friends (16.4% vs. 14.4%), and social media (23.0% vs. 19.6%) as preferred information sources. Descriptively, students attending a Gymnasium more often selected parents (33.8%) and social media (22.8%), whereas students from other school types more frequently selected teachers (22.0%) and physicians (21.6%).

Adolescents who did not currently use cigarettes, e-cigarettes, or waterpipes were more likely than current users to select parents (31.8% vs. 15.8%), teachers (19.1% vs. 10.0%), and physicians (19.4% vs. 11.0%) as preferred information sources. In contrast, current users more frequently selected friends (20.0% vs. 15.0%) and social media (24.4% vs. 21.1%) as preferred sources (Table 1).

**Table 1.** Preferred Information Sources on the Health Risks of Waterpipes, Cigarettes, and e-Cigarettes Among Adolescents (%) With 95% Confidence Intervals, Stratified by Age, Gender, School Type, and Nicotine Use (N=21,841)

Source of information	Total	Age ≤14 yrs	Age 15–17 yrs	Female	Male	Gymnasium	Other school types	Non-use of cigarettes, e-cigarettes, waterpipes	Current use of cigarettes, e-cigarettes, waterpipes
% (95% CI)									
Parents	30.2 (29.6-30.8)	32.8 (32.0-33.5)	23.6 (22.6-24.7)	31.8 (31.0-32.7)	28.7 (27.8-29.6)	33.8 (33.0-34.7)	27.1 (26.2-28.0)	31.8 (31.1-32.4)	15.8 (14.2-17.5)
Social media	21.5 (20.9-22.0)	19.4 (18.7-20.0)	26.9 (25.8-28.1)	23.0 (22.3-23.8)	19.6 (18.8-20.4)	22.8 (22.0-23.5)	20.3 (19.5-21.2)	21.2 (20.6-21.7)	24.4 (22.5-26.4)
Physicians	18.6 (18.1-19.2)	18.5 (17.9-19.2)	18.9 (17.9-19.9)	18.4 (17.7-19.1)	18.7 (18.0-19.5)	21.6 (20.9-22.4)	16.0 (15.3-16.8)	19.4 (19.9-20.0)	11.0 (9.7-12.5)
Teachers	18.3 (17.7-18.8)	18.2 (17.6-18.8)	18.4 (17.4-19.4)	20.2 (19.5-21.0)	16.3 (15.6-17.0)	22.0 (21.2-22.7)	15.0 (14.3-15.8)	19.1 (18.6-19.7)	10.0 (8.7-11.5)
Friends	15.5 (15.0-16.0)	15.0 (14.4-15.6)	16.6 (15.7-17.6)	16.4 (15.7-17.1)	14.4 (13.7-15.1)	15.9 (15.3-16.6)	15.1 (14.4-15.8)	15.0 (14.5-15.5)	20.0 (18.2-21.9)
Apps/websites	11.1 (10.6-11.5)	10.9 (10.4-11.4)	11.4 (10.6-12.2)	10.8 (10.2-11.4)	11.2 (10.6-11.8)	11.9 (11.4-12.5)	10.3 (9.7-10.9)	11.1 (10.7-11.6)	10.3 (9.0-11.8)
Public posters	10.5 (10.1-10.9)	10.3 (9.8-10.8)	11.0 (10.3-11.8)	10.7 (10.2-11.3)	10.1 (9.5-10.7)	12.1 (11.6-12.7)	9.1 (8.5-9.7)	10.7 (10.3-11.1)	8.4 (7.2-9.7)
Health insurance providers	6.6 (6.3-6.9)	6.6 (6.2-7.0)	6.6 (6.0-7.2)	5.7 (5.3-6.1)	7.3 (6.8-7.8)	7.2 (6.7-7.6)	6.1 (5.6-6.6)	6.7 (6.4-7.1)	5.3 (4.4-6.5)
Sports clubs	6.3 (6.0-6.7)	6.3 (6.0-6.7)	6.3 (5.7-7.0)	4.7 (4.3-5.1)	7.8 (7.3-8.3)	6.9 (6.5-7.4)	5.8 (5.4-6.3)	6.2 (5.9-6.6)	7.4 (6.2-8.7)

Footnotes/Legend. Percentages indicate the proportion of adolescents reporting each source of information. Age groups. ≤14 years vs. 15–17 years. School types. Gymnasium (higher academic track) vs. other school types. Nicotine use.: Non-use of cigarettes, e-cigarettes, waterpipes vs. Current use of cigarettes, e-cigarettes, waterpipes.

### Associations Between Preferred Information Sources, Sociodemographic Characteristics, and Nicotine-Product Use

Adjusted multilevel logistic regression models indicated that age, gender, school type, and nicotine-use status were associated with preferred information sources (Table 2). Compared with adolescents aged 9-14 years, those aged 15-17 years were more likely to select posters in public spaces (aOR = 1.17, 95% CI [1.05–1.29], p = 0.003), friends (aOR = 1.10, 95% CI [1.00–1.20], p = 0.048), teachers (aOR = 1.15, 95% CI [1.05–1.26], p = 0.002), physicians (aOR = 1.14, 95% CI [1.04–1.24], p < 0.001), and social media (aOR = 1.56, 95% CI [1.44–1.69], p < 0.001). In contrast, older adolescents were less likely to select parents as a preferred information source (aOR = 0.71, 95% CI [0.66–0.77], p < 0.001); see Table 2.

**Table 2.** Adjusted Odds Ratios (aOR) and 95% Confidence Intervals (CI) for Adolescents’ Preferred Information Sources About the Health Risks of Waterpipes, Cigarettes, and e-Cigarettes, by Age, Gender, School Type, and Nicotine Use Status

Source of information	Age	Gender	School type	Nicotine use
	15 to 17 years (reference: up to 14 years)	Male (reference: Female)	Gymnasium (reference: lower academic schools)	Current use of cigarettes, e-cigarettes, waterpipes (reference: Non-use of cigarettes, e-cigarettes, waterpipes)
Sports club	n.s.	aOR=1,71 (1,53-1,92) p<0.001	aOR= 1,26 (1,12-1,42) p<0.001	n.s.
Health insurance companies	n.s.	aOR=1,32 (1,18-1,47) p<0.001	aOR=1,21 (1,07-1,36) p=0.002	aOR=0,72 (0,57-0,89) p=0.003
Posters in public spaces	aOR=1,17 (1,05-1,29) p=0.003	n.s.	aOR=1,37 (1,24-1,50) p<0.001	aOR=0,74 (0,62-0,88) p=0.001
Apps/websites	n.s.	n.s	aOR=1,20 (1,09-1,32), p<0.001	n.s.
Friends	aOR=1,10 (1,00-1,20) p=0.048	aOR=0,87 (0,81-0,95) p=0.001	aOR= 1,09 (1,01-1,19) p=0.036	aOR=1,37 (1,20-1,55) p<0.001
Teachers	aOR=1,15 (1,05-1,25) p=0.002	aOR=0,77 (0,72-0,83), p<0.001	aOR=1,52 (1,41-1,67) p<0.001	aOR=0,46 (0,39-0,54) p<0.001
Physician	aOR=1,14 (1,04-1,24) p<0.001	n.s.	aOR=1,41 (1,30-1,53), p<0.001	aOR=0,50 (0,42-0,59) p<0.001
Social media	aOR=1,56 (1,44-1,69) p<0.001	aOR=0,82 (0,76-0,87) p<0.001	aOR=1,21 (1,12-1,31) p<0.001	n.s.
Parents	aOR=0,71 (0,66-0,77) p<0.001	aOR=0,87 (0,82-0,93), p<0.001	aOR=1,26 (1,18-1,35) p<0.001	aOR=0,44 (03,9-0,51) p<0.001

Footnotes/Legend. Age groups. ≤14 years vs. 15–17 years. School types. Gymnasium (higher academic track) vs. other school types. Nicotine use.: Non-use of cigarettes, e-cigarettes, waterpipes vs. Current use of cigarettes, e-cigarettes, waterpipes.

Compared with girls, boys were more likely to select sports clubs (aOR = 1.71, 95% CI [1.53–1.91], p < 0.001) and health insurance providers (aOR = 1.32, 95% CI [1.18–1.47], p < 0.001) as preferred information sources. Conversely, boys were less likely to select friends (aOR = 0.87, 95% CI [0.81–0.94], p = 0.001), teachers (aOR = 0.77, 95% CI [0.72–0.83], p < 0.001), social media (aOR = 0.82, 95% CI [0.76–0.87], p < 0.001), and parents (aOR = 0.87, 95% CI [0.82–0.93], p < 0.001).

Students attending a Gymnasium were more likely than students attending other school types to select sports clubs (aOR = 1.26, 95% CI [1.12–1.42], p < 0.001), health insurance providers (aOR = 1.21, 95% CI [1.07–1.36], p = 0.002), posters in public spaces (aOR = 1.37, 95% CI [1.24–1.50], p < 0.001), apps or websites (aOR = 1.20, 95% CI [1.09–1.32], p < 0.001), friends (aOR = 1.09, 95% CI [1.01–1.19], p = 0.036), teachers (aOR = 1.52, 95% CI [1.41–1.67], p < 0.001), physicians (aOR = 1.41, 95% CI [1.30–1.53], p < 0.001), social media (aOR = 1.21, 95% CI [1.12–1.31], p < 0.01), and parents (aOR = 1.26, 95% CI [1.18–1.35], p < 0.001). This pattern suggests a broader stated openness to multiple information sources among students in the higher academic track.

Current users of at least one assessed tobacco or nicotine product were more likely than non-users to select friends as a preferred source of information (aOR = 1.37, 95% CI [1.20–1.55], p < 0.001). In contrast, current users were less likely to select health insurance providers (aOR = 0.72, 95% CI [0.57–0.89], p = 0.003), posters in public spaces (aOR = 0.74, 95% CI [0.62–0.88], p = 0.001), teachers (aOR = 0.46, 95% CI [0.39–0.54], p < 0.001), physicians (aOR = 0.50, 95% CI [0.42–0.59], p < 0.001), and parents (aOR = 0.44, 95% CI [0.39–0.51], p < 0.001). No significant associations were found between current nicotine-product use and selection of sports clubs, apps or websites, or social media.

### Household Smoking Environment and Parental Information Preference

Regarding household smoking behavior, the findings indicate that adolescents living in households where smoking occurs exclusively outside the home have significantly higher odds of selecting their parents as a preferred source of information compared to those from smoke-free households (OR = 1.20, 95% CI [1.12, 1.29], p < 0.001). In contrast, the findings revealed no significant effect for adolescents living in households where smoking takes place inside the home (OR = 1.04, 95% CI [0.93, 1.16], p = 0.499).

### Information Refusal

**Figure 2.**
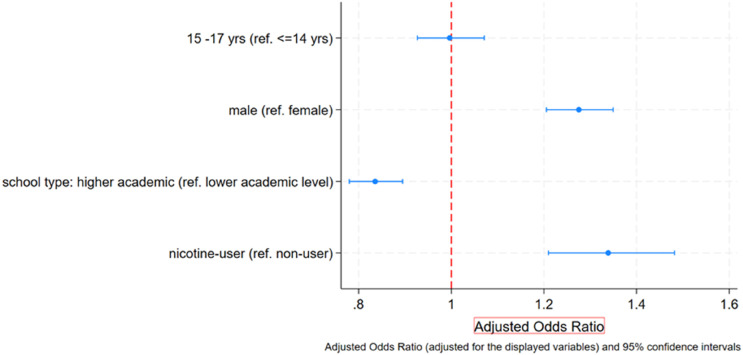
Factors associated with refusing information: adjusted multilevel model results

In adjusted multilevel models accounting for class-level clustering, information refusal was associated with gender, school type, and nicotine-use status, whereas age showed no significant association. Boys were more likely than girls to indicate that they did not want information (aOR = 1.28, 95% CI 1.21–1.35, p < 0.001). Students attending a Gymnasium were less likely than students attending other school types to select this option (aOR = 0.83, 95% CI 0.78–0.89, p < 0.001). Current users of at least one assessed tobacco or nicotine product were more likely to refuse information than non-users (aOR = 1.34, 95% CI 1.21–1.48, p < 0.001) (Figure 2).

A significant interaction between school type and nicotine-use status was observed (aOR = 1.45, 95% CI 1.18–1.79, p = 0.001). Among non-users, students attending a Gymnasium had a lower predicted probability of information refusal than students from other school types (38.4% vs. 43.3%). In contrast, among current users, Gymnasium students had a higher predicted probability of information refusal than students from other school types (50.8% vs. 47.0%).

The interaction between male gender and nicotine-use status suggested that the increase in information refusal associated with nicotine use was attenuated among boys (aOR = 0.77, 95% CI 0.63–0.94, p = 0.01). In other words, the combined association of male gender and nicotine use with information refusal was smaller than would be expected from the independent associations of these factors.

## Discussion

The findings from this large-scale school-based sample of more than 21,000 adolescents highlight pronounced social and behavioral gradients in preferred sources of information about the health risks of waterpipes, cigarettes, and e-cigarettes. Parents, social media, and physicians were the most frequently selected sources overall, but preferences differed substantially by age, gender, school type, and current nicotine-product use. Such preferences should not be interpreted as evidence of trust in a source, actual exposure to information from that source, engagement with the information provided, or the effectiveness of messages delivered through that source.

### Age-Specific Communication Pathways

Older adolescents were more likely to select peers, teachers, physicians, and social media as preferred information sources, whereas younger adolescents more often selected parents. This pattern is consistent with developmental models emphasizing increasing autonomy from parents and greater peer orientation during mid-to-late adolescence.^14^ For prevention practice, these findings identify sources that younger and older adolescents report as acceptable or desirable for receiving information. Whether messages delivered through these sources achieve sufficient reach, credibility, engagement, or preventive effects remains to be established.

### Gender Differences in Information Preferences

Gender-specific patterns further emphasize the need for differentiated prevention strategies. Boys were more likely to select sports clubs and health insurance providers, whereas girls more frequently selected parents, teachers, friends, and social media. This may indicate that sport-related settings may warrant further investigation as communication settings for boys, while rational and media-based approaches may warrant further investigation among girls. Sports clubs and health insurance providers may therefore warrant consideration when developing and evaluating gender-sensitive communication strategies for male adolescents. However, the present findings do not demonstrate that these sources would be trusted or effective.

However, these findings must be interpreted cautiously. The higher selection of sports clubs among boys may reflect greater participation in organized sports rather than a genuine preference for this communication channel. Future research should incorporate measures of actual exposure, institutional membership, and message engagement to disentangle access from preference.

### Educational Gradients and Health Inequalities

Students attending the higher academic track showed greater stated openness to a broad range of information sources in adjusted analyses. This finding is consistent with broader evidence on social gradients in health communication and health literacy.^15,16^ It may indicate that conventional communication approaches are more acceptable or accessible to socially advantaged adolescents. From a tobacco-control perspective, this is important because standard prevention formats may be less likely to reach adolescents in lower academic tracks adequately. Without targeted and low-barrier strategies for students from lower academic tracks, preventive communication may fail to reach groups at elevated risk and may inadvertently contribute to the persistence of tobacco-related health inequalities.

### Information Preferences Among Nicotine-Product Users

Adolescents who currently used cigarettes, e-cigarettes, or waterpipes displayed a distinct information profile. They were more likely to select friends and less likely to select authoritative or institutional sources such as parents, teachers, physicians, health insurance providers, and posters in public spaces. This pattern is consistent with theories of selective exposure and cognitive dissonance, which propose that individuals tend to avoid information that threatens their current behaviors.^17^ Similar avoidance of formal health information has been observed among adolescents in previous studies.^18^

These findings suggest that conventional institution-based prevention strategies may be less acceptable to adolescents who already use nicotine products. Peer-led, youth-oriented, and non-moralizing digital approaches may warrant further investigation for engaging adolescents who currently use nicotine products. However, their acceptability, credibility, reach, and effectiveness should be evaluated in appropriately designed intervention studies.

### Information Refusal as a Prevention Barrier

Particularly concerning is the finding that boys, students from lower academic tracks, and current nicotine-product users were more likely to reject health information altogether. Information avoidance has increasingly been recognized as a barrier to effective prevention, especially among individuals engaging in health-risk behaviors.^19^ In the present study, adolescents at elevated risk of tobacco- and nicotine-related harm appeared less receptive to conventional prevention efforts.

Several psychological and social mechanisms may contribute to information refusal. Previous research has linked health-information avoidance to risk perception, information overload, negative emotions, social norms, and low perceived source credibility.^20^ Adolescents’ willingness to engage with health information may also depend on their health literacy and trust in the platform or messenger.^21,22^ Among current nicotine-product users, avoiding information that conflicts with existing behavior or beliefs may additionally reduce cognitive dissonance. However, these mechanisms were not directly assessed in the present study and should therefore be regarded as plausible explanations rather than established mediators.

These findings underscore the importance of low-barrier, non-stigmatizing, and relevance-oriented communication strategies that reduce perceived judgment and enhance personal relevance among disengaged groups.

The observed interactions further suggest that information refusal is not uniformly distributed across risk groups. Among non-users, students attending a Gymnasium had a lower predicted probability of information refusal than students from other school types. Among current users, however, Gymnasium students had a higher predicted probability of information refusal. This pattern may indicate that current nicotine-product use modifies the relationship between educational track and receptiveness to health information. Further research is needed to understand the mechanisms underlying this finding, including possible roles of risk perception, perceived social norms, autonomy, and prior exposure to prevention messages.

### Implications for Tobacco and Nicotine Prevention

Taken together, the findings support testing multimodal approaches to adolescent tobacco and nicotine prevention. The preferred sources identified in this study may inform the selection of communication settings and messengers. Strategies using family, school, healthcare, peer, community, and digital sources should therefore be developed with adolescents and evaluated with respect to reach, credibility, engagement, knowledge, risk perception, and behavioural outcome. Prevention strategies should also be equity-oriented. Communication formats should be designed to reach students from lower academic tracks and adolescents who are less likely to actively seek health information. This may require co-designed messages, low-barrier digital formats, outreach in non-academic settings, and communication that emphasizes autonomy, credibility, and relevance rather than fear-based or moralizing appeals.

### Strengths and Limitations

A major strength of this study is its large school-based sample of more than 21,000 adolescents from grades 5 to 10 across 14 German federal states, drawn from an established monitoring system. The broad geographic coverage and class-based recruitment enabled robust subgroup analyses by age, gender, school type, and nicotine-use status. Moreover, directly assessing adolescents’ preferred information channels provides valuable target-group-specific evidence for the development of tailored tobacco and nicotine prevention strategies.

Several limitations should be considered. First, although weighting was applied for age, gender, and school type, participation at the school and class level was voluntary. Selection bias therefore cannot be ruled out, and the sample should not be interpreted as strictly representative of all adolescents in Germany. Second, the cross-sectional design precludes causal inference. It remains unclear whether preference for certain information sources influences nicotine-related behavior, whether nicotine use shapes information preferences, or whether both are influenced by other factors.

Third, the study assessed preferred information sources, not actual exposure to prevention messages or the effectiveness of messages delivered through these channels. Preference should therefore not be equated with preventive impact. Fourth, the item assessing preferred information sources had previously been pretested for comprehensibility among adolescents and young adults aged 15-34 years, but it was not explicitly pretested among the younger target population of the present survey. Although selected questionnaire items were phrased in simpler and more age-appropriate language, differences in comprehension, particularly among younger students, cannot be excluded. In addition, formal criterion validity and test–retest reliability were not assessed. The findings should therefore be interpreted as self-reported channel preferences rather than as scores derived from a formally validated psychometric instrument. Fifth, because the outcome allowed multiple responses, selected information sources were not mutually exclusive. Fifth, some reported preferences may reflect differential access rather than genuine preference. For example, higher selection of sports clubs among boys may partly reflect higher participation in organized sports. Finally, the outcome combined waterpipes, cigarettes, and e-cigarettes in a single question. We were therefore unable to determine whether adolescents prefer different information sources for different tobacco or nicotine products. Although the survey assessed whether smoking occurred in the home environment, it did not identify which household member smoked. Consequently, this measure cannot be interpreted as a direct assessment of parental smoking status. Future studies should assess the tobacco- and nicotine-use status of parents and other household members separately and examine how this influences the content and perceived credibility of family-based health communication. One further limitation of this study is the absence of an a priori power analysis to justify the sample size. As the study examining preferred sources of health risk information on cigarettes, e-cigarettes, and waterpipes among adolescents based on a large convenience sample drawn from a pre-existing dataset, no formal calculation was performed prior to analysis. However, the dataset was weighted and adjusted to reflect the underlying population structure, which enhances the representativeness of the findings. It should also be noted that in large-scale observational studies such as this one, the need for an a priori power calculation is generally less critical, as the extensive sample size typically provides sufficient statistical power to detect meaningful effects. Nonetheless, the absence of a formal power analysis should be considered when interpreting the statistical precision of the results.

The study assessed preferred information sources rather than trust in these sources, actual exposure to health information, engagement with specific messages, or communication effectiveness. A source that is frequently selected may not necessarily be perceived as credible or produce changes in knowledge, risk perception, intentions, or behavior. Conversely, a less preferred source may still be effective when messages are appropriately designed and delivered. The findings should therefore be interpreted as evidence on stated channel preferences and as a basis for developing and testing communication strategies, rather than as evidence that the identified sources constitute effective prevention channel.

## Conclusion

This study provides large-scale evidence on adolescents’ stated preferences for sources of health risk information about cigarettes, e-cigarettes, and waterpipes in Germany. Preferences varied by age, gender, school type, and nicotine-use status. Adolescents who currently used nicotine products were less likely to prefer conventional adult-led or institutional sources and more likely to select friends or refuse information altogether. These findings may inform the development of differentiated and multimodal communication strategies, but they do not establish trust, exposure, engagement, or effectiveness. Future studies should evaluate whether interventions delivered through preferred sources improve reach, credibility, risk understanding, and prevention outcomes, particularly among adolescents who are difficult to engage through conventional approaches.

## Data Availability

The data that support the findings of this study are available from RH (co-author) upon reasonable request.*
